# Electrolyte Imbalances and Metabolic Emergencies in Obesity: Mechanisms and Clinical Implications

**DOI:** 10.3390/diseases13030069

**Published:** 2025-02-24

**Authors:** Iulia Najette Crintea, Alexandru Cristian Cindrea, Ovidiu Alexandru Mederle, Cosmin Iosif Trebuian, Romulus Timar

**Affiliations:** 1 Department of Surgery, “Victor Babes” University of Medicine and Pharmacy, 300041 Timisoara, Romania; iulia.crintea@umft.ro (I.N.C.); alexandru.cindrea@umft.ro (A.C.C.); trebuian.cosmin@umft.ro (C.I.T.); 2 Emergency Department, Emergency Clinical Municipal Hospital, 300079 Timisoara, Romania; 3 Department of Anesthesia and Intensive Care, Emergency County Hospital, 320210 Resita, Romania; 4 “Pius Brinzeu” Emergency County Hospital, 300723 Timisoara, Romania; timar.romulus@umft.ro; 5 Second Department of Internal Medicine, “Victor Babes” University of Medicine and Pharmacy, 300041 Timisoara, Romania

**Keywords:** obesity, electrolyte imbalances, metabolic emergencies, obesity management, insulin resistance, metabolic acidosis

## Abstract

Electrolyte imbalances are a frequently overlooked yet critical component of obesity-related metabolic dysfunction, contributing to an increased risk of cardiovascular disease, kidney impairment, and metabolic emergencies such as diabetic ketoacidosis (DKA), hyperosmolar hyperglycemic state (HHS), and acute kidney injury (AKI). These disturbances arise from insulin resistance, chronic inflammation, hormonal dysregulation, and renal dysfunction, leading to sodium retention, potassium depletion, and deficiencies in calcium and magnesium homeostasis. Managing electrolyte imbalances is essential in obesity management, as imbalances exacerbate hypertension, metabolic acidosis, neuromuscular complications, and insulin resistance. This review explores the pathophysiology of electrolyte disturbances in obesity and their impact on fluid balance, acid–base status, and metabolic health. Effective management strategies include individualized electrolyte monitoring, dietary sodium restriction, potassium supplementation, vitamin D and magnesium correction, and pharmacologic interventions targeting renin–angiotensin–aldosterone system (RAAS) activity and insulin resistance. Additionally, lifestyle interventions, including dietary modification, weight loss strategies, and hydration optimization, play a key role in preventing metabolic complications. Future research should investigate the long-term impact of electrolyte imbalances in obesity, the role of emerging therapies, and how lifestyle interventions can optimize electrolyte homeostasis and metabolic outcomes. A personalized, multidisciplinary approach integrating endocrinology, nephrology, and clinical nutrition is essential to improving the prevention and management of electrolyte imbalances in obese individuals.

## 1. Electrolyte Imbalances in Obesity: Mechanisms and Impact

Obesity has emerged as a significant global health concern, contributing to various metabolic and systemic complications [1]. Among these, electrolyte imbalances play a crucial yet often overlooked role in metabolic dysfunction. Electrolytes—including sodium, potassium, calcium, magnesium, and phosphate—are fundamental for maintaining physiological functions such as nerve conduction, muscle contraction, acid–base balance, and fluid regulation. Disruptions in their balance contribute to metabolic emergencies and systemic dysfunctions [2,3].

Electrolyte disturbances are frequently observed in individuals with obesity due to a combination of insulin resistance, chronic inflammation, hormonal dysregulation, and renal dysfunction. Sodium retention, potassium depletion, and deficiencies in calcium and magnesium homeostasis exacerbate conditions such as hypertension, metabolic acidosis, neuromuscular complications, and cardiovascular risks [4,5,6,7]. The pathophysiology of these imbalances is complex and multifactorial, involving dysregulated renal handling of electrolytes, altered endocrine signaling, and increased oxidative stress [8,9].

Understanding the relationship between obesity and electrolyte disturbances is essential for developing effective management strategies. This review synthesizes current evidence, exploring the mechanisms driving electrolyte imbalances in obesity and discussing targeted interventions, including dietary modifications, pharmacological approaches, and lifestyle changes to optimize metabolic health [10,11]. Furthermore, the clinical consequences of electrolyte imbalances, particularly their role in worsening metabolic conditions such as insulin resistance and cardiovascular dysfunction, are highlighted [12,13].

With the increasing prevalence of obesity, addressing electrolyte imbalances as part of comprehensive metabolic management is imperative. Future research should further investigate the long-term impact of electrolyte disturbances, potential advancements in electrolyte-targeting therapies, and the role of bariatric interventions in restoring electrolyte homeostasis.

Electrolytes are essential for maintaining cell function, fluid balance, nerve conduction and metabolic stability. Their homeostasis is tightly regulated by the renal, endocrine, and cardiovascular systems. However, in obesity, systemic metabolic dysregulation creates a predisposition for electrolyte imbalances, leading to pathological consequences. The interaction among insulin resistance, chronic inflammation, and renal dysfunction contributes to altered sodium, potassium, calcium, magnesium, and phosphate levels, with downstream effects on fluid balance, acid–base balance, and cardiovascular function [14,15,16].

In contrast to individuals with a stable metabolic profile, obese patients frequently develop chronic electrolyte dysregulation due to an altered hormonal environment characterized by hyperinsulinemia, dysregulation of the renin–angiotensin–aldosterone system (RAAS), and increased oxidative stress. These disorders exacerbate metabolic instability, increasing susceptibility to severe complications such as hypertension, metabolic acidosis, and kidney damage. In addition, excess adipose tissue acts as an endocrine organ, modulating the release of proinflammatory cytokines that further impair electrolyte transport and renal function [17,18,19].

Given these challenges, it is essential to study the specific electrolyte imbalances associated with obesity, their underlying mechanisms, and their clinical impact. This chapter will provide an in-depth review of how obesity influences sodium, potassium, calcium, magnesium, and phosphate homeostasis, with an emphasis on their implications for metabolic emergencies such as diabetic ketoacidosis (DKA), hyperosmolar hyperglycemic state (HHS), and acute kidney injury (AKI).

Obesity predisposes individuals to significant electrolyte disorders, primarily affecting sodium, potassium, calcium, and magnesium homeostasis. These imbalances occur due to a combination of insulin resistance, hormonal disorders, and renal dysfunction, contributing to metabolic instability and cardiovascular risk. Table 1 provides a comprehensive overview of primary electrolyte imbalances in obesity, summarizing their pathophysiological mechanisms and clinical implications. Understanding these disorders is essential for developing specific interventions to prevent complications and optimize metabolic health in obese patients.

### 1.1. Sodium Dysregulation in Obesity

Sodium is an electrolyte essential in maintaining extracellular fluid (ECF) volume and regulating blood pressure. In people with obesity, alterations in sodium handling can disrupt these processes. Research indicates that obesity is associated with an increase in ECF volume relative to intracellular fluid (ICF), leading to decreased serum sodium concentrations. This imbalance is linked to hypertension and abdominal obesity, components of the metabolic syndrome [24].

High sodium intake has been implicated in the development of obesity and metabolic disorders. Elevated sodium consumption may stimulate thirst and appetite, leading to increased energy intake, a significant factor in obesity. Studies have shown a positive association between sodium intake and measures of obesity, such as body mass index (BMI) and waist circumference [25].

Recent studies have explored the mechanisms by which high sodium intake contributes to obesity and associated metabolic disorders. One proposed pathway involves leptin resistance. Leptin, a hormone derived from adipose tissue, regulates energy balance by suppressing appetite and increasing energy expenditure. However, excessive sodium intake has been linked to impaired leptin signaling, leading to increased food intake and reduced energy expenditure. Animal studies have shown that high salt intake may induce leptin resistance, thereby promoting weight gain and adiposity [26]. Additionally, high sodium intake has been associated with insulin resistance and the development of metabolic syndrome. The exact mechanisms remain under investigation, but it is suggested that excessive sodium may alter insulin signaling pathways, contributing to impaired glucose metabolism [27].

Given the established link between high sodium intake, obesity, and hypertension, public health guidelines have emphasized the importance of reducing dietary sodium consumption. The World Health Organization recommends a daily sodium intake of less than 2 g to mitigate the risk of cardiovascular diseases. However, global sodium consumption often exceeds these recommendations, underscoring the need for public health interventions to promote dietary modifications [28]. Recent initiatives have advocated the use of salt substitutes, which replace some of the sodium chloride with potassium chloride, as a strategy to reduce sodium intake without compromising taste. Studies have shown that such substitutes can effectively reduce blood pressure and may offer a feasible approach to reducing dietary sodium at the population level [29].

Hypernatremia, characterized by elevated serum sodium levels exceeding 145 mmol/L, is a significant electrolyte disturbance that can have profound implications, particularly in obese individuals. The interaction between excessive body weight and sodium imbalance exacerbates the risk of developing hypernatremia and its associated complications [30].

In obese individuals, the regulation of water and sodium balance is often compromised. The increased adipose tissue alters the distribution of body water, leading to a relative decrease in total body water per unit of body weight. This reduction makes obese individuals more susceptible to water depletion, especially during periods of inadequate fluid intake or excessive water loss [31]. Moreover, obesity is frequently associated with impaired thirst mechanisms and reduced sensitivity to osmoreceptors, which can delay the recognition of dehydration and the subsequent drive to consume water. These factors contribute to the development of hypernatremia in the obese population [32].

Elevated serum sodium levels can lead to significant neurological disorders. The hypertonic state caused by hypernatremia causes the removal of water from brain cells, leading to dehydration and cell shrinkage. This cell contraction can cause rupture of cerebral blood vessels, increasing the risk of intracranial hemorrhage. Clinically, patients may present with altered mental status, ranging from confusion and lethargy to seizures and coma in severe cases [33]. Neuromuscular irritability is also common, manifesting as twitching, hyperreflexia, or muscle spasticity. These neurologic manifestations emphasize the critical need for prompt recognition and management of hypernatremia to prevent irreversible brain damage [34,35].

The kidneys play an essential role in maintaining sodium and water homeostasis. In hypernatremia, the renal concentrating mechanism is challenged to conserve water [36]. However, in obese individuals, renal function may already be compromised due to obesity-related glomerulopathy, characterized by glomerular hyperfiltration and subsequent proteinuria. This renal impairment affects the ability of the kidneys to concentrate urine efficiently, exacerbating water loss and perpetuating hypernatremia. In addition, the use of certain medications in obese patients, such as loop diuretics for the treatment of hypertension, may further impair renal concentrating ability, increasing the risk of hypernatremia [37].

Hypernatremia induces a hypertonic state, leading to intracellular dehydration and a subsequent increase in plasma osmolality. This hyperosmolar state triggers the release of vasopressin (antidiuretic hormone), promoting water reabsorption in the kidneys to dilute elevated sodium levels. At the same time, vasopressin induces vasoconstriction, increasing peripheral vascular resistance and consequently blood pressure [38]. In obese people, who are already at increased risk of hypertension due to factors such as increased sympathetic activity and insulin resistance, the additional vasoconstriction mediated by vasopressin may precipitate or aggravate hypertensive states. This increase in blood pressure increases the risk of cardiovascular events, including myocardial infarction and stroke [39].

Obesity is often accompanied by insulin resistance and hyperinsulinemia. Hypernatremia can exacerbate insulin resistance, as high sodium levels have been shown to impair insulin signaling pathways. This impairs glucose uptake by cells, leading to hyperglycemia. The resulting hyperglycemia stimulates additional insulin release, creating a vicious cycle that can culminate in the development or worsening of type 2 diabetes [40]. In addition, the combination of hypernatremia and insulin resistance may promote oxidative stress and inflammation, contributing to endothelial dysfunction and increased risk of atherosclerosis [41].

Management of hypernatremia in obese patients requires a nuanced approach that addresses both the correction of sodium imbalance and the underlying factors that contribute to its development [42]. Gradual correction of serum sodium is essential to prevent the risk of cerebral edema, a serious complication arising from rapid changes in osmolarity. The general recommendation is to reduce serum sodium levels by no more than 8 mmol/L over a 24 h period. This cautious approach allows the osmotic adaptive mechanisms of the brain to adjust, minimizing the risk of cell swelling and increased intracranial pressure [43].

In addition to careful fluid management, addressing lifestyle factors is essential. Encouraging adequate water intake is essential for preventing dehydration and maintaining optimal hydration status. Dietary modifications, such as reducing sodium intake by limiting processed and sodium-rich foods, can help prevent the recurrence of hypernatremia. Implementing a balanced diet rich in fruits, vegetables, and whole grains not only helps regulate sodium but also supports overall cardiovascular health [44]. Regular monitoring of serum electrolytes is vital in the management of hypernatremia, particularly in hospitalized obese patients who may have multiple comorbidities and are at an increased risk of electrolyte imbalance. Frequent assessment allows timely detection of sodium level deviations and facilitates prompt intervention, thereby reducing the risk of complications [45]. In cases where hypernatremia is secondary to an underlying endocrine disorder, such as diabetes insipidus, appropriate pharmacologic interventions should be initiated. For example, desmopressin can be administered for the management of central diabetes insipidus by reducing urine output and promoting water retention, thereby correcting hypernatremia [46].

### 1.2. Potassium Dysregulation

Potassium is an essential electrolyte involved in numerous physiological processes including nerve conduction, muscle contraction and acid–base balance. Its homeostasis is tightly regulated by renal function, hormonal control (particularly insulin and aldosterone), and dietary intake. In obesity, potassium dysregulation is frequently observed, mainly due to insulin resistance, chronic inflammation, and renal changes. In an euglycemic state, insulin facilitates potassium transport into muscle and liver cells, thereby maintaining serum potassium levels within a narrow range [14]. However, in obesity-induced insulin resistance, insulin’s ability to transport intracellular potassium is impaired, leading to relative hypokalemia in the extracellular compartment. This contributes to altered cardiac excitability and neuromuscular function, increasing the risk of arrhythmias and muscle weakness [47].

In addition to insulin resistance, obese people often exhibit hyperaldosteronism, a compensatory mechanism in response to the expanded extracellular fluid volume. Aldosterone increases renal potassium excretion, further aggravating potassium depletion in obese patients [48]. Studies suggest that chronic activation of the renin–angiotensin–aldosterone system (RAAS) in obesity exacerbates hypokalemia by increasing urinary potassium loss [15]. This dysregulation is particularly evident in metabolically unhealthy obese individuals, who have greater electrolyte disturbances compared to metabolically healthy counterparts [49].

In addition, dietary factors contribute to potassium imbalances in obesity. A Western diet, usually low in potassium-rich foods (such as fruits and vegetables) and high in sodium, disrupts potassium–sodium homeostasis. Epidemiologic data show that potassium deficiency is more prevalent in obese populations, correlating with increased risks of hypertension, insulin resistance, and cardiovascular disease [42,43]. Given the importance of potassium in metabolic and cardiovascular stability, addressing dietary insufficiencies and optimizing potassium intake is essential in the management of obesity [50].

Hypokalemia in obesity is a multifactorial condition with profound consequences for cardiovascular and metabolic health. Cardiac complications associated with hypokalemia include ventricular arrhythmias, prolonged QT intervals, and increased risk of sudden cardiac death, especially in obese patients with pre-existing cardiovascular disease. Electrophysiologic instability caused by potassium deficiency predisposes patients to life-threatening arrhythmias, which are further exacerbated by hypertension and diabetes [51].

In addition, the interaction between potassium imbalance and metabolic dysregulation has a significant impact on glucose homeostasis. Hypokalemia has been linked to reduced insulin secretion, impairing pancreatic β-cell function and worsening hyperglycemia in obese individuals. This relationship establishes a vicious cycle in which obesity-induced insulin resistance leads to potassium loss, further exacerbating metabolic instability [52]. Clinical studies have shown that people with low potassium levels have a higher prevalence of type 2 diabetes and metabolic syndrome, emphasizing the interdependent relationship between electrolyte imbalances and metabolic dysfunction [53].

In addition, potassium depletion impairs vascular function, contributing to hypertension and endothelial dysfunction. Potassium plays a critical role in vasodilatation by modulating vascular smooth muscle tone, and its deficiency leads to increased vascular resistance and increased blood pressure. This is of particular concern in obese individuals, who already have an increased burden of hypertension and cardiovascular morbidity [54,55].

Finally, potassium imbalances may also contribute to renal complications in obesity. Hypokalemia induces tubulointerstitial injury, exacerbating obesity-related chronic kidney disease (CKD). Potassium depletion has been associated with glomerular hyperfiltration, increased sodium reabsorption, and increased oxidative stress, all of which contribute to progressive renal dysfunction [56]. Addressing potassium imbalance in obese individuals is therefore a crucial strategy to mitigate the risk of cardiovascular and renal complications.

Potassium dysregulation in obesity is caused by a combination of insulin resistance, hyperaldosteronism, dietary inadequacy, and renal changes. These disturbances contribute to significant cardiac, metabolic, and renal complications, emphasizing the need for early detection and intervention [57]. Future research should explore specific potassium supplementation strategies and dietary modifications to mitigate these risks, particularly in obese individuals with coexisting metabolic disorders.

### 1.3. Calcium and Magnesium Dysregulation

Calcium and magnesium are vital electrolytes involved in bone metabolism, neuromuscular function, and enzyme reactions. Their homeostasis is mainly regulated by hormonal control, particularly parathyroid hormone (PTH), vitamin D, and calcitonin. In obesity, chronic low-grade inflammation and hormonal imbalances contribute to disruption of calcium and magnesium levels, predisposing individuals to metabolic complications [58].

Parathyroid hormone (PTH) dysregulation is commonly seen in obesity. Studies have shown that obese people often have elevated PTH levels even in the presence of normal or low calcium levels, a condition known as secondary hyperparathyroidism [59]. This response is thought to result from vitamin D deficiency, which is highly prevalent in obesity due to increased sequestration of vitamin D in adipose tissue, reduced bioavailability, and impaired hepatic conversion to its active form. Vitamin D is essential for calcium absorption in the intestines; thus, its deficiency contributes to hypocalcemia and compensatory elevation of PTH, further disrupting calcium metabolism [60].

In addition, chronic systemic inflammation, a feature of obesity, exacerbates calcium and magnesium imbalances. Proinflammatory cytokines, such as tumor necrosis factor-alpha (TNF-α) and interleukin-6 (IL-6), have been shown to interfere with calcium and magnesium absorption in the intestines and kidneys [61]. These inflammatory mediators also impair osteoblastic function and increase bone resorption, which can lead to osteopenia and osteoporosis in obese individuals. In addition, inflammatory cytokines have been linked to renal calcium and magnesium loss, further contributing to electrolyte deficiencies [62].

The metabolic implications of hypocalcemia and hypomagnesemia in obesity are substantial, affecting glucose homeostasis, cardiovascular health, and neuromuscular function. Magnesium is a cofactor for tyrosine kinase activity in insulin receptors, which facilitates glucose uptake into target tissues. Hypomagnesemia has been associated with reduced insulin sensitivity and increased risk of type 2 diabetes. Studies suggest that magnesium supplementation may improve insulin resistance and glucose tolerance in obese people [23]. Similarly, calcium plays a role in insulin secretion from pancreatic β-cells, and hypocalcemia may affect insulin release and increase the risk of metabolic dysfunction [63].

Both hypocalcemia and hypomagnesemia are linked to cardiovascular complications in obesity. Magnesium deficiency is a known risk factor for hypertension and arrhythmias as it modulates vascular tone and endothelial function [64]. Hypocalcemia, on the other hand, has been implicated in QT prolongation, increasing the risk of sudden cardiac death. The interaction among calcium, magnesium, and vascular function emphasizes the importance of maintaining an optimal electrolyte balance in obese individuals [65].

Despite the increased bone mass often observed in obese people due to greater mechanical loading, obesity has paradoxically been associated with reduced bone quality and an increased risk of fractures. This is partly due to calcium and magnesium deficiencies, which affect bone mineralization and remodeling. The role of increased PTH and vitamin D deficiency in this process further emphasizes the complex relationship between obesity and skeletal health [66].

Calcium and magnesium imbalances in obesity result from a combination of hormonal dysregulation, chronic inflammation, insulin resistance, and dietary inadequacies. These disturbances contribute to metabolic dysfunction, cardiovascular disease, and skeletal disorders, emphasizing the clinical importance of early detection and intervention. Addressing vitamin D deficiency, optimizing dietary intake of calcium and magnesium, and alleviating inflammation may provide beneficial strategies to improve metabolic health in obese individuals.

## 2. Pathophysiology of Electrolyte Imbalances in Obese Patients

Electrolyte imbalances in obese patients occur due to a combination of factors, including insulin resistance, chronic inflammation, hormonal dysregulation, and renal dysfunction. These disorders affect the transport and balance of essential electrolytes such as sodium, potassium, calcium, and magnesium, increasing the risk of metabolic and cardiovascular complications [67,68]. Insulin resistance in particular directly influences sodium and potassium transport across cell membranes, contributing to fluid retention and hypertension [69]. Chronic inflammation and renal dysfunction further exacerbate these imbalances by impairing the mechanisms responsible for electrolyte reabsorption and excretion [70]. The following sections will explore in detail how insulin resistance influences sodium and potassium homeostasis in obese individuals.

### 2.1. Insulin Resistance and Electrolyte Transport

The Na^+^/K^+^-ATPase pump is a key regulator of electrolyte transport, facilitating intracellular uptake of potassium (K^+^) and extracellular excretion of sodium (Na^+^) to maintain electrochemical gradients. In insulin-resistant states, this process is impaired due to deregulated insulin signaling, which reduces ATPase activity and decreases K^+^ uptake into cells. This dysfunction contributes to a predisposition to hyperkalemia, which can disrupt cardiac electrophysiology and lead to arrhythmias [71]. In addition, defective extrusion of Na^+^ due to insulin resistance leads to intracellular sodium accumulation, increasing vascular resistance and blood pressure. These changes contribute to the development of obesity-related hypertension, a condition that significantly increases cardiovascular risk [48].

In addition to cellular transport dysfunction, insulin resistance disrupts sodium handling in the kidney. Under normal physiologic conditions, insulin enhances sodium reabsorption in proximal and distal renal tubules, helping to maintain intravascular volume. However, in insulin-resistant individuals, this mechanism becomes exaggerated, leading to excessive sodium retention, increased plasma volume, and systemic hypertension [21].

**Figure 1:**
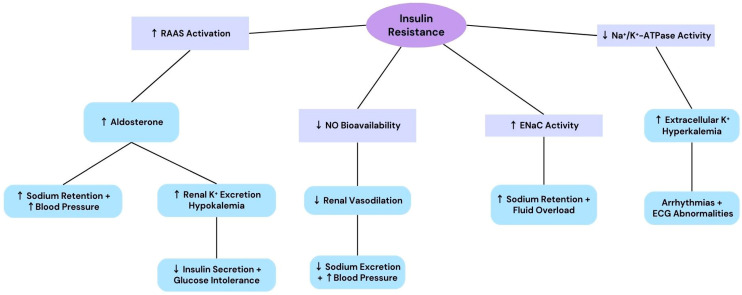
Sodium and Potassium Dysregulation in Insulin Resistance. Legend for Abbreviations: RAAS: Renin–Angiotensin–Aldosterone System; NO: Nitric Oxide; ENaC: Epithelial Sodium Channel; ECG: Electrocardiogram.

Insulin resistance disrupts sodium and potassium homeostasis through multiple interconnected mechanisms, as illustrated in Figure 1. Activation of the renin–angiotensin–aldosterone system (RAAS) is a central pathway, leading to increased aldosterone secretion, which promotes sodium retention and increases blood pressure. Concomitantly, RAAS overactivation increases renal excretion of potassium, predisposing to hypokalemia, which further impairs insulin secretion and worsens glucose intolerance. The reduced bioavailability of nitric oxide (NO) contributes to vascular dysfunction by impairing renal vasodilatation, thereby limiting sodium excretion and exacerbating hypertension. In addition, increased epithelial sodium channel (ENaC) activity amplifies sodium retention and fluid overload. On the other hand, decreased Na^+^/K^+^-ATPase activity leads to extracellular potassium accumulation, causing hyperkalemia, which increases the risk of arrhythmias and electrocardiographic (ECG) abnormalities. This figure integrates these pathways to demonstrate the complex interplay between sodium and potassium dysregulation in insulin resistance and its systemic impact [72,73].

### 2.2. Chronic Inflammation and Oxidative Stress

Obesity induces a chronic inflammatory state, where adipose tissue functions as a secretory organ releasing pro-inflammatory mediators. These cytokines and chemokines, including tumor necrosis factor-alpha (TNF-α), interleukin-6 (IL-6), and monocyte chemoattractant protein-1 (MCP-1), contribute to endothelial dysfunction, insulin resistance, and renal impairment, all of which disrupt electrolyte homeostasis [74].

One of the key mechanisms through which chronic inflammation influences electrolyte imbalance is renal sodium retention. TNF-α and IL-6 have been shown to upregulate epithelial sodium channel (ENaC) expression in the renal tubules, thereby increasing sodium reabsorption and extracellular fluid volume expansion, a process that promotes hypertension and volume overload. In addition, inflammation suppresses natriuretic peptide activity, further impairing sodium excretion [75].

The excessive lipotoxicity and mitochondrial dysfunction observed in obesity lead to the overproduction of reactive oxygen species (ROS), which further propagates renal endothelial dysfunction and tubular damage. ROS decreases the availability of nitric oxide (NO), a vasodilator essential for renal autoregulation, leading to increased renal vascular resistance and impaired sodium excretion. The reduction in NO also promotes glomerular hyperfiltration, which accelerates nephron loss and worsens electrolyte handling [76]. Oxidative stress additionally affects potassium regulation by modifying renal Na^+^/K^+^-ATPase pump activity. Elevated oxidative stress promotes tubular potassium loss, predisposing individuals to hypokalemia, while simultaneously contributing to an intracellular shift of potassium, potentially resulting in intermittent hyperkalemia under acute stress conditions [77,78].

The persistent inflammatory and oxidative burden in obesity significantly accelerates kidney damage, predisposing individuals to chronic kidney disease (CKD) and worsening electrolyte disturbances [79]. This state is further characterized by the following:

- Enhanced sodium retention and hypertension, increasing cardiovascular risk.
- Impaired potassium excretion, leading to fluctuations between hypokalemia and hyperkalemia.
- Calcium and magnesium deficiencies, promoting metabolic and neuromuscular dysfunctions.

Chronic inflammation and oxidative stress serve as critical mediators of obesity-induced renal dysfunction and electrolyte disturbances, altering sodium, potassium, calcium, and magnesium homeostasis. The interplay among cytokine activity, oxidative damage, and renal sodium retention not only exacerbates hypertension but also increases the risk of diabetes progression and cardiovascular complications. Future therapeutic interventions targeting anti-inflammatory pathways and oxidative stress reduction could offer promising strategies in mitigating the impact of electrolyte imbalances in obese individuals.

### 2.3. Hormonal Dysregulation in Obesity

Obesity is associated with multiple hormonal disorders, which play an important role in the regulation of electrolyte homeostasis, fluid balance, and metabolic processes. These disturbances involve not only the renin–angiotensin–aldosterone system (RAAS) but also changes in the parathyroid hormone (PTH), vitamin D metabolism, and glucocorticoid activity, contributing to sodium retention, potassium loss, potassium loss, and calcium/magnesium imbalances. Unlike previous sections that focus on RAAS-mediated sodium and potassium changes, this section will emphasize PTH dysregulation, vitamin D deficiency, and the role of cortisol in obesity-induced electrolyte disorders [80].

PTH is a key regulator of calcium homeostasis, influencing bone resorption, renal excretion of calcium, and intestinal absorption of calcium. In obesity, a state of secondary hyperparathyroidism (SHPT) is frequently observed, even in the presence of normal or slightly reduced serum calcium levels. This condition is largely attributed to chronic vitamin D deficiency, which results from its sequestration in adipose tissue, decreased hepatic conversion to its active form (1,25-dihydroxyvitamin D), and obesity-related inflammatory cytokine activity that suppresses renal 1α-hydroxylase [81]. Elevated PTH levels in obesity drive increased renal phosphate retention, which, in turn, suppresses calcitriol synthesis, further impairing calcium absorption. Additionally, excessive PTH secretion stimulates osteoclastic activity, increasing bone turnover and paradoxically leading to low bone quality and an increased fracture risk despite the higher mechanical load in obese individuals. The simultaneous effects of high PTH and chronic inflammation promote vascular calcification, contributing to hypertension and cardiovascular disease in obese patients [82].

Another factor contributing to calcium-phosphate dysregulation in obesity is fibroblast growth factor 23 (FGF-23), a hormone that is elevated in metabolic disorders. FGF-23 inhibits renal phosphate reabsorption and suppresses calcitriol production, exacerbating phosphate retention and calcium imbalances. This suggests that obesity-related hyperphosphatemia may play a role in metabolic bone disease and cardiovascular calcification beyond the effects of PTH alone [83].

Magnesium plays an essential role in cellular metabolism, neuromuscular function, and glucose regulation. Hypomagnesemia is common in obesity and contributes significantly to insulin resistance and metabolic dysfunction. Chronic inflammation, driven by IL-6 and TNF-α, promotes renal magnesium wasting, while hyperinsulinemia itself accelerates magnesium excretion by increasing glomerular filtration. The depletion of magnesium negatively impacts Na^+^/K^+^-ATPase function, worsening intracellular potassium loss and further impairing insulin signaling [84]. Magnesium is also required for the activation of tyrosine kinase in the insulin receptor, meaning that low magnesium levels reduce insulin sensitivity, creating a vicious cycle that perpetuates obesity-related metabolic dysfunction. Furthermore, magnesium deficiency is linked to an increased risk of cardiac arrhythmia, endothelial dysfunction, and hypertension, as it enhances vascular smooth muscle contraction and impairs nitric oxide bioavailability [85].

Obese individuals with magnesium depletion also exhibit higher levels of oxidative stress, which exacerbates mitochondrial dysfunction and contributes to lipotoxicity and metabolic inflammation. Given its essential role in energy homeostasis and glucose metabolism, correcting magnesium deficiency may represent a therapeutic strategy for improving metabolic outcomes in obesity [86].

### 2.4. Cortisol Dysregulation and Its Impact on Electrolyte Balance

The hypothalamic-pituitary–adrenal (HPA) axis is frequently dysregulated in obesity, leading to increased cortisol production and reduced clearance. While circulating cortisol levels may appear normal in many obese individuals, local cortisol activity is enhanced due to increased expression of 11β-hydroxysteroid dehydrogenase type 1 (11β-HSD1) in adipose tissue. This enzyme converts inactive cortisone into active cortisol, promoting lipid accumulation, insulin resistance, and systemic inflammation [87].

Excess cortisol activates mineralocorticoid receptors in the kidney, mimicking aldosterone’s effects and leading to sodium retention and potassium loss. This mechanism contributes to fluid overload, hypertension, and hypokalemia, further exacerbating metabolic syndrome [88]. Cortisol also suppresses osteoblast function, promoting bone resorption and increasing calcium excretion, which, in combination with PTH dysregulation, worsens obesity-related bone disease. Furthermore, excess glucocorticoids disrupts circadian rhythms and appetite regulation, driving increased food intake and adiposity, further perpetuating the cycle of obesity and hormonal dysfunction. Chronic cortisol elevation is also associated with altered gut microbiota composition, which may further impact electrolyte absorption and renal handling [89].

The interplay among PTH, vitamin D, magnesium, and cortisol imbalances significantly worsens metabolic and cardiovascular health in obese individuals. The combined effects of secondary hyperparathyroidism, hypomagnesemia, and hypercortisolism contribute to a state of systemic electrolyte dysregulation, promoting hypertension, insulin resistance, osteoporosis, and increased cardiovascular risk.

### 2.5. Renal Dysfunction and Obesity

Obesity imposes a significant burden on renal function through a combination of metabolic, hemodynamic, and inflammatory mechanisms. The kidneys play a central role in maintaining electrolyte balance, fluid homeostasis, and acid–base equilibrium, and their dysfunction in obesity leads to a cascade of metabolic and cardiovascular complications. As adipose tissue expands, it exerts both direct and indirect effects on the renal system, altering sodium and water handling, promoting oxidative stress, and impairing structural integrity. These alterations drive the progression of chronic kidney disease (CKD), placing obese individuals at heightened risk of kidney dysfunction even in the absence of diabetes or hypertension [37].

One of the earliest manifestations of obesity-related kidney dysfunction is glomerular hyperfiltration, a compensatory response to increased metabolic demands. The rise in renal plasma flow and glomerular filtration rate (GFR) initially appears protective, maintaining sodium and fluid balance [90]. However, prolonged hyperfiltration places excessive mechanical stress on glomerular capillaries, leading to endothelial injury and podocyte dysfunction. Over time, this leads to structural damage, including glomerulosclerosis and tubulointerstitial fibrosis, which contribute to the progressive loss of renal function. The development of obesity-related glomerulopathy (ORG) is characterized by podocyte loss, mesangial expansion, and segmental sclerosis, ultimately resulting in proteinuria and CKD progression [91].

In addition to structural changes, obesity profoundly alters sodium handling within the nephron. Enhanced sodium reabsorption occurs primarily in the proximal tubules due to increased sodium-glucose cotransporter 2 (SGLT2) activity, which is exacerbated by insulin resistance. The upregulation of epithelial sodium channels (ENaC) and heightened activation of the RAAS further contribute to sodium retention, leading to extracellular volume expansion and hypertension [92]. Despite this, some obese individuals exhibit increased urinary sodium excretion in later disease stages, reflecting compensatory pressure natriuresis. However, this mechanism is often insufficient to counteract the persistent sodium retention driven by hormonal and neural influences [20].

Potassium imbalances in obesity arise due to a combination of renal dysregulation, insulin resistance, and RAAS overactivation. As detailed in Section 3.1, Na^+^/K^+^-ATPase dysfunction contributes to intracellular potassium depletion, but additional mechanisms, including aldosterone-mediated potassium excretion and chronic metabolic acidosis, further exacerbate potassium disturbances. Clinical management of potassium imbalances in obese individuals should focus on individualized potassium monitoring, dietary potassium optimization, and careful use of diuretics to prevent hypokalemia-induced cardiac arrhythmias [56]. This paradoxical effect results in potassium fluctuations that contribute to metabolic instability, neuromuscular dysfunction, and arrhythmias. At the same time, obesity-related renal dysfunction disrupts acid–base homeostasis, predisposing individuals to metabolic acidosis. Reduced ammonium excretion and increased acid generation from adipose tissue metabolism exacerbate systemic acid retention, which further impairs insulin sensitivity and muscle function [92].

Inflammation and oxidative stress are key drivers of obesity-induced kidney dysfunction. Adipose tissue releases pro-inflammatory cytokines (TNF-α, IL-6, MCP-1), causing endothelial dysfunction, vascular permeability, and tubular injury, leading to nephron loss. Oxidative stress worsens renal damage by promoting lipid peroxidation, mitochondrial dysfunction, and reduced nitric oxide (NO) availability, resulting in vasoconstriction and impaired sodium excretion. Hypoxia from adipose tissue compression on renal vessels further accelerates kidney injury [93].

The impact of obesity on kidney function extends beyond electrolyte imbalances, increasing the risk of nephrolithiasis and AKI. Obese individuals are predisposed to kidney stone formation due to a combination of hypercalciuria, hyperoxaluria, and hypocitraturia, which promote calcium crystal aggregation. The increased excretion of uric acid, often seen in insulin-resistant states, further acidifies urine and increases the likelihood of uric acid stone formation [94]. Furthermore, episodes of AKI are more frequent in obese patients due to hemodynamic instability, heightened inflammatory responses, and lipotoxicity-induced tubular damage. Unlike non-obese individuals, those with excess adiposity exhibit impaired renal recovery following AKI, leading to progressive nephron loss and increased susceptibility to CKD [95].

Given the strong association between obesity and renal dysfunction, early screening and intervention are critical. Weight reduction strategies, including lifestyle modifications and pharmacologic interventions, have been shown to mitigate renal hyperfiltration and reduce proteinuria. Emerging therapies, such as SGLT2 inhibitors, have demonstrated promise in attenuating glomerular hypertension, improving sodium handling, and preserving renal function in obese patients. Dietary interventions aimed at reducing sodium intake and increasing potassium and magnesium consumption may help counteract electrolyte imbalances, while anti-inflammatory and antioxidant therapies offer potential avenues for preventing obesity-related renal complications [96].

Understanding the mechanisms underlying obesity-induced kidney dysfunction is essential for developing targeted therapeutic strategies. By addressing the interplay among metabolic, inflammatory, and hemodynamic factors, clinicians can better manage the renal complications of obesity and reduce the burden of CKD in this high-risk population.

## 3. Electrolyte Imbalances and Metabolic Emergencies in Obesity

Obesity increases the risk of metabolic emergencies due to electrolyte disturbances, fluid imbalances, and impaired homeostasis [97]. The combination of insulin resistance, chronic inflammation, and renal dysfunction predisposes individuals to acute conditions such as diabetic ketoacidosis (DKA), hyperosmolar hyperglycemic state (HHS), and acute kidney injury (AKI) [53]. However, given the scope of this review, we provide a brief overview of HHS and AKI while emphasizing their connections to electrolyte imbalances in obesity.

### 3.1. Diabetic Ketoacidosis (DKA) and Electrolyte Shifts

Diabetic ketoacidosis (DKA) is a severe metabolic emergency exacerbated by insulin resistance and obesity, leading to profound electrolyte disturbances [98,99]. Hyperglycemia-driven osmotic diuresis depletes sodium, potassium, and phosphate, contributing to dehydration, acidosis, and systemic instability [100].

Potassium depletion is critical, often masked initially but unmasked upon insulin administration, necessitating close monitoring to prevent fatal arrhythmias [101]. Sodium disturbances, including pseudohyponatremia and hypernatremia, further complicate fluid management and require careful correction to avoid cerebral edema [102,103]. Hypophosphatemia, though often overlooked, can impair cellular energy metabolism and exacerbate muscle weakness and respiratory failure [104]. Given the overlapping metabolic derangements in DKA, hyperosmolar hyperglycemic state (HHS), and acute kidney injury (AKI), fluid resuscitation and electrolyte repletion strategies should be carefully individualized [101,105]. Obese patients may experience more severe metabolic dysfunction due to preexisting renal impairment and chronic inflammation, necessitating a nuanced therapeutic approach to balance fluid correction and prevent complications such as pulmonary edema or prolonged ketoacidosis resolution [106].

Early recognition and targeted intervention are crucial to optimizing outcomes in obese patients with DKA, emphasizing close electrolyte monitoring, gradual fluid replacement, and prevention of systemic deterioration [101].

### 3.2. Hyperosmolar Hyperglycemic State (HHS)

HHS is characterized by extreme hyperglycemia and severe dehydration without significant ketoacidosis, often resulting in hyperosmolarity, neurological dysfunction, and multi-organ failure [106]. The absence of significant ketoacidosis in HHS delays clinical recognition, increasing the severity of electrolyte imbalances and the risk of cerebral edema if fluid replacement is too rapid [107].

Both conditions lead to severe potassium depletion, although potassium shifts in HHS are less pronounced due to the absence of acidosis. However, osmotic diuresis and insulin deficiency exacerbate potassium loss, necessitating early monitoring to prevent life-threatening cardiac instability [51,108]. Sodium imbalances in HHS can be misleading due to pseudohyponatremia, while true hypernatremia occurs as dehydration worsens, necessitating gradual correction to avoid neurological complications [102,107].

HHS also increases the risk of acute kidney injury (AKI) due to prolonged hypovolemia, further complicating electrolyte management [109]. Given the overlap of metabolic abnormalities in HHS and DKA, fluid resuscitation and electrolyte correction should be carefully individualized to prevent complications such as pulmonary edema or prolonged ketoacidosis resolution [110,111].

Early recognition and targeted intervention are crucial in both conditions, emphasizing gradual osmolar correction, potassium and sodium monitoring, and the prevention of systemic deterioration [108].

### 3.3. Acute Kidney Injury (AKI) and Electrolyte Disruptions

AKI in obesity is driven by hemodynamic alterations, oxidative stress, and inflammation, often leading to sodium retention, potassium dysregulation, and an increased risk of chronic kidney disease (CKD) [112].

Sodium imbalances in AKI vary between fluid overload due to hyperaldosteronism and sodium depletion during acute ischemic episodes, impacting cardiovascular and renal function [113]. Potassium disturbances range from hyperkalemia in impaired excretion to hypokalemia in polyuric phases, requiring careful electrolyte monitoring [114]. Magnesium and phosphate imbalances exacerbate oxidative stress and endothelial dysfunction, further complicating renal outcomes [62,63].

Acid–base disorders such as metabolic acidosis and alkalosis influence insulin resistance, fluid homeostasis, and cardiovascular stability, worsening metabolic instability in obese individuals with AKI [115]. Given the complex interplay between obesity and electrolyte dysregulation, early recognition, individualized fluid therapy, and electrolyte correction are crucial for optimizing outcomes and preventing CKD progression [108].

## 4. Clinical Implications and Management Strategies

Electrolyte imbalances in obese individuals have significant clinical implications, contributing to an increased risk of metabolic emergencies, cardiovascular complications, and renal dysfunction [116]. Given the complex interplay among insulin resistance, chronic inflammation, altered hormonal regulation, and renal impairment, a comprehensive approach is required for effective management [117]. The following sections discuss recent advancements in electrolyte-targeting therapies, the metabolic consequences of bariatric surgery, the effects of weight-loss medications, and the potential for precision medicine in electrolyte homeostasis.

### 4.1. Emerging Pharmacologic Approaches in Electrolyte Management

Recent pharmacological advancements have introduced novel therapeutic agents for correcting electrolyte disturbances in obese individuals. Sodium-glucose co-transporter-2 (SGLT2) inhibitors have demonstrated benefits in promoting renal sodium excretion and improving glycemic control, thereby mitigating hypertension and extracellular fluid overload [118]. Additionally, potassium binders such as patiromer and sodium zirconium cyclosilicate have shown efficacy in controlling chronic hyperkalemia, particularly in patients with obesity and insulin resistance [119]. Furthermore, renin–angiotensin–aldosterone system (RAAS) modulators play a crucial role in improving sodium and potassium balance while reducing the risk of obesity-related hypertension and kidney dysfunction [120].

Micronutrient supplementation is also essential for maintaining metabolic stability. Magnesium and vitamin D correction not only improve bone mineral density but also enhance insulin sensitivity and vascular function, preventing secondary complications of obesity-induced electrolyte disturbances. These therapies contribute to a more targeted and individualized approach to electrolyte homeostasis [121].

### 4.2. Bariatric Surgery and Its Effects on Electrolyte Homeostasis

Bariatric surgery, particularly Roux-en-Y gastric bypass (RYGB) and sleeve gastrectomy, induces profound metabolic changes that affect electrolyte balance [122]. Postoperative hypokalemia, hypomagnesemia, and vitamin D deficiencies are frequently reported, necessitating close monitoring and tailored supplementation. Rapid weight loss following surgery can alter sodium and potassium excretion, increasing the risk of dehydration and orthostatic hypotension [123]. Additionally, metabolic adaptations, such as increased secretion of gut hormones, improve glucose metabolism but may also disrupt calcium and phosphate homeostasis. Therefore, electrolyte monitoring and individualized supplementation protocols are essential in postoperative management to prevent complications such as bone mineral loss, cardiac arrhythmias, and neuromuscular dysfunction [124].

### 4.3. Impact of Weight-Loss Medications on Electrolyte Balance

Pharmacologic weight-loss interventions contribute significantly to electrolyte regulation. Glucagon-like peptide-1 (GLP-1) receptor agonists, including semaglutide and liraglutide, enhance potassium uptake into cells and improve insulin sensitivity, which can influence potassium homeostasis in obese patients [125]. SGLT2 inhibitors, in addition to glycemic control, induce natriuresis and osmotic diuresis, making them beneficial for individuals with hypertension and fluid overload [126]. Bupropion/naltrexone therapy, used for weight loss, has demonstrated metabolic benefits by reducing inflammation, which indirectly impacts electrolyte balance by modulating renal sodium handling and potassium regulation [127].

### 4.4. Precision Medicine and Individualized Electrolyte Management

The integration of precision medicine in electrolyte management is an emerging field that offers tailored therapeutic strategies based on genetic, metabolic, and clinical profiles. Pharmacogenomics is being utilized to predict patient-specific responses to RAAS inhibitors and diuretics, thereby reducing the risk of adverse electrolyte imbalances. Additionally, machine-learning models are being developed to optimize electrolyte correction strategies by analyzing real-time metabolic data. These approaches enable a more personalized and adaptive method of managing electrolyte disturbances in obesity-related metabolic dysfunction [128].

### 4.5. Lifestyle Interventions and Electrolyte Homeostasis

The relationship among electrolyte balance, body composition, and energy metabolism is a crucial yet often overlooked aspect of obesity management. Electrolytes such as sodium, potassium, calcium, and magnesium play essential roles in fluid balance, muscle function, and metabolic regulation, all of which are fundamental to maintaining energy expenditure and body composition [129]. In obesity, disruptions in electrolyte homeostasis contribute to metabolic inefficiency, impaired glucose metabolism, and cardiovascular dysfunction, further exacerbating weight gain and obesity-related complications. Lifestyle interventions, including dietary modifications, exercise programs, and weight loss strategies, offer an effective means to restore electrolyte balance while improving overall metabolic health [51].

Electrolyte disturbances in obesity are not simply consequences of metabolic dysfunction; they actively influence body composition, energy storage, and thermogenesis. Chronic sodium retention leads to extracellular fluid expansion, increasing total body weight and contributing to hypertension and cardiovascular stress [126]. Potassium depletion, often observed in insulin-resistant individuals, affects muscle contraction, nerve conduction, and glucose utilization, impairing physical performance and metabolic rate [127]. Calcium and magnesium imbalances have been associated with reduced muscle mass, mitochondrial dysfunction, and impaired insulin sensitivity, leading to a decline in basal metabolic rate (BMR) and overall energy efficiency [123]. Addressing these imbalances through targeted lifestyle interventions may optimize body composition, muscle function, and metabolic flexibility in obese individuals.

Nutritional interventions represent a primary approach to correcting electrolyte imbalances and supporting metabolic health. Sodium intake has a direct impact on fluid retention and hypertension, and obese individuals benefit from sodium restriction, which can reduce extracellular volume, lower blood pressure, and improve weight loss outcomes. A diet rich in potassium-containing foods, including fruits, vegetables, legumes, and nuts, has been shown to enhance insulin sensitivity, promote muscle function, and counteract sodium-induced fluid retention [129]. Calcium and vitamin D intake are critical in obese populations, given the high prevalence of secondary hyperparathyroidism and bone mineral density loss. Adequate dietary calcium from dairy products, fortified foods, and supplements may help maintain neuromuscular function and prevent metabolic bone disease. Magnesium plays an essential role in glucose metabolism, mitochondrial energy production, and cardiovascular function; supplementation may help reduce insulin resistance and improve muscle strength in obese individuals [130].

Physical activity has a profound influence on electrolyte regulation, fluid balance, and metabolic efficiency. Exercise increases sodium and potassium turnover through heightened renal excretion and sweat loss, necessitating proper hydration strategies in obese individuals who engage in structured physical activity [131]. Increased Na^+^/K^+^-ATPase activity during exercise enhances intracellular potassium retention, reducing the risk of hyperkalemia while supporting neuromuscular function. Exercise also stimulates renal sodium excretion, counteracting the chronic sodium retention and extracellular fluid expansion observed in obesity [92]. Regular resistance and endurance training improve bone remodeling and calcium metabolism, reducing the risk of osteopenia and impaired skeletal muscle function. Magnesium-dependent ATP production is also enhanced with aerobic and resistance training, optimizing energy metabolism and reducing fatigue in overweight and obese individuals. However, given the altered fluid and electrolyte dynamics in obesity, exercise regimens must be paired with individualized hydration and electrolyte replacement strategies to prevent muscle cramps, arrhythmias, and exercise-induced fatigue [132].

Weight loss interventions, including caloric restriction and bariatric surgery, have significant effects on electrolyte balance and metabolic adaptation. Bariatric surgery, particularly gastric bypass and sleeve gastrectomy, alters sodium, potassium, calcium, and magnesium absorption, leading to an increased risk of postoperative electrolyte deficiencies. Lifelong nutritional monitoring and supplementation are essential in surgically treated obese individuals to prevent malnutrition and electrolyte disturbances. Caloric restriction, particularly in low-calorie diets and ketogenic diets, influences renal sodium handling, leading to transient natriuresis and fluid shifts [133]. The resulting initial weight loss may be attributed to sodium and water excretion, necessitating proper fluid and sodium intake adjustments to prevent dehydration, orthostatic hypotension, and metabolic alkalosis. Protein-rich diets have been shown to enhance potassium retention and support muscle preservation, making them a critical component of metabolically healthy weight loss programs. Emerging research on intermittent fasting suggests that prolonged fasting periods may alter sodium and potassium homeostasis, particularly in individuals with preexisting insulin resistance [134]. Optimizing electrolyte intake during fasting periods is necessary to sustain energy metabolism and prevent metabolic stress.

Given the profound effects of weight loss, exercise, and dietary changes on electrolyte homeostasis, it is imperative that personalized electrolyte monitoring and correction strategies are integrated into obesity management plans. The interplay among electrolyte regulation, energy metabolism, and weight regulation underscores the necessity of a multidisciplinary approach, incorporating clinical nutrition, nephrology, endocrinology, and exercise physiology to optimize long-term health outcomes in obese individuals. Addressing electrolyte imbalances not only mitigates metabolic complications but also enhances body composition, physical performance, and energy efficiency, making it a critical aspect of comprehensive obesity treatment strategies.

A multidisciplinary approach involving lifestyle interventions, dietary modifications, pharmacologic strategies, and regular electrolyte monitoring is required to mitigate the long-term complications of obesity-associated electrolyte disturbances. Addressing fluid balance, insulin resistance, and renal function remains central to the prevention and treatment of metabolic emergencies in obese patients, ensuring improved quality of life and long-term health stability.

### 4.6. Future Directions

Given the increasing prevalence of obesity and its associated metabolic disturbances, future research should focus on evaluating the long-term effects of electrolyte-targeting therapies, expanding the role of precision medicine in electrolyte monitoring, and further understanding the metabolic consequences of bariatric surgery. Additionally, developing targeted interventions to prevent electrolyte imbalances in patients undergoing pharmacologic weight-loss therapies will be essential in improving clinical outcomes and reducing the burden of metabolic complications in obesity.

By incorporating these advancements into clinical practice, clinicians can optimize electrolyte stability, prevent metabolic emergencies, and improve the long-term health outcomes of obese individuals.

## 5. Conclusions

Electrolyte imbalances are involved in the metabolic complications of obesity, contributing to cardiovascular disease, kidney dysfunction, and metabolic emergencies such as DKA, HHS, and AKI. Insulin resistance caused by obesity, chronic inflammation, and kidney dysfunction disrupts homeostasis of sodium, potassium, calcium, and magnesium, increasing morbidity and mortality. Clinically, targeted electrolyte management is essential. Sodium restriction, potassium monitoring, vitamin D and magnesium supplementation, and acid–base correction are key strategies for preventing complications. Personalized treatment approaches are needed to balance fluid therapy, hormonal disorders, and organ function in obese people.

Future research should explore the long-term impact of electrolyte imbalances, new pharmacological interventions, and the role of bariatric surgery in correcting metabolic dysfunction. A multidisciplinary approach that integrates nephrology, endocrinology, and metabolic medicine is crucial for optimizing electrolyte homeostasis and improving clinical outcomes in obesity.

## Figures and Tables

**Table 1:** Electrolyte Imbalances in Obesity: Mechanisms and Clinical Consequences. This table summarizes the major electrolyte disturbances observed in obese individuals, highlighting their underlying mechanisms and clinical consequences. RAAS = Renin–Angiotensin–Aldosterone System, CKD = Chronic Kidney Disease, PTH = Parathyroid Hormone, FGF-23 = Fibroblast Growth Factor 23.

Electrolyte	Primary Imbalance in Obesity	Mechanisms	Clinical Consequences
Sodium (Na^+^)	Hypernatremia, sodium retention	RAAS overactivation, insulin resistance, reduced natriuresis [16]	Hypertension, volume overload, increased CKD risk [12]
Potassium (K^+^)	Hypokalemia (more common), hyperkalemia (less common)	Impaired Na^+^/K^+^-ATPase function, aldosterone-driven K^+^ loss, insulin resistance [20]	Arrhythmias, muscle weakness, glucose intolerance [21]
Calcium (Ca^2+^)	Hypocalcemia, secondary hyperparathyroidism	Vitamin D deficiency, increased PTH secretion, FGF-23 elevation [4]	Osteoporosis, vascular calcification, hypertension [8]
Magnesium (Mg^2+^)	Hypomagnesemia	Insulin resistance, renal wasting, inflammation-induced depletion [22]	Increased insulin resistance, arrhythmias, endothelial dysfunction [23]
